# Rates and risk factors for amputation in people with diabetes in Japan: a historical cohort study using a nationwide claims database

**DOI:** 10.1186/s13047-021-00474-8

**Published:** 2021-04-09

**Authors:** Masanori Kaneko, Kazuya Fujihara, Mayuko Yamada Harada, Taeko Osawa, Masahiko Yamamoto, Masaru Kitazawa, Yasuhiro Matsubayashi, Takaho Yamada, Hiroyasu Seida, Satoru Kodama, Hirohito Sone

**Affiliations:** 1 Department of Internal Medicine, Niigata University Faculty of Medicine, 1-754 Asahimachi, Niigata, Niigata 951-8510 Japan; 2 JMDC Inc., Tokyo, Japan

**Keywords:** Lower limb amputation, Risk factor, HbA1c, Age, Asian people

## Abstract

**Background:**

The prevalence of diabetes is rising, and diabetes develops at a younger age in East Asia. Although lower limb amputation negatively affects quality of life and increases the risk of cardiovascular events, little is known about the rates and predictors of amputation among persons with diabetes from young adults to those in the “young-old” category (50–72 y).

**Methods:**

We analyzed data from a nationwide claims database in Japan accumulated from 2008 to 2016 involving 17,288 people with diabetes aged 18–72 y (mean age 50.2 y, HbA1c 7.2%). Amputation occurrence was determined according to information from the claims database. Cox regression model identified variables related to lower limb amputation.

**Results:**

The mean follow-up time was 5.3 years, during which time 16 amputations occurred (0.17/1000 person-years). Multivariate Cox regression analysis showed that age (hazard ratio [HR] 1.09 [95% confidence intervals] 1.02–1.16, *p* = 0.01) and HbA1c (HR 1.46 [1.17–1.81], *p* < 0.01) were independently associated with amputations. Compared with those aged < 60 years with HbA1c < 8.0%, the HR for amputation was 27.81 (6.54–118.23) in those aged ≥60 years and HbA1c ≥8.0%.

**Conclusions:**

Age and HbA1c were associated with amputations among diabetic individuals, and the rates of amputation were significantly greater in those ≥60 years old and with HbA1c ≥8.0%.

**Supplementary Information:**

The online version contains supplementary material available at 10.1186/s13047-021-00474-8.

## Background

The prevalence of type 2 diabetes is rising in Asia, characterized by onset at a relatively young age and low body mass index (BMI) compared with Western countries [1–3]. Diabetes-related complications, including lower-extremity amputation, are a significant cause of increased mortality among people with diabetes and have substantial economic consequences [4–6]. Lower limb amputation not only markedly reduces patients’ quality of life [7] but also increases risks of cardiovascular events and death [8, 9]. Gujral et al. reported that the age- and sex-adjusted incidence rate of lower limb amputation for those with diabetes mellitus of Asian ethnic origin was estimated to be 0.34 (95% confidence interval (CI), 0.11–1.07) cases per 1000 person-years compared to 1.42 (1.26–1.59) in Caucasians [10]. However, since diabetes develops at a younger age in East Asia, a study directly exploring this issue among Japanese diabetic individuals from young adults to those in the “young-old” category is needed.

Longer exposure to glycated haemoglobin A1c (HbA1c) levels above target was associated with increased risk of both micro- and macrovascular complications [11, 12]. However, a recent meta-analysis evaluating risk factors for lower extremity amputation in patients with diabetic foot ulcers showed that HbA1c levels do not affect the incidence of amputation [13]. Studies included in that meta-analysis were only on patients with diabetic foot ulcers who had received recent intensive treatment [13], suggesting that the impact of HbA1c on amputation may have been underestimated.

Age is an established independent risk factor for atherosclerosis and macrovascular complications in people with diabetes [14, 15]. However, a meta-analysis showed that age was not related to amputation in diabetic foot ulcer patients [13]. In most studies, the average participants are middle aged or older [10, 16, 17]. Thus, the impact of age on lower limb amputation is not clear among Japanese diabetic individuals ranging from young adults to the “young-old” (50–72 y). Therefore, we aimed to determine the rates of lower limb amputations and risk factors for lower limb amputations in Japanese people with diabetes among young adults to those in the “young-old” category.

## Methods

### Database

Data were obtained from a nationwide claims database that was provided by JMDC Inc. Details of the database were described previously [15, 18]. Briefly, the JMDC collects claims data from approximately 6,000,000 people who belong to a health insurance provider for company employees and their dependents. This database also has data on annual medical check-ups for some of its participants that include data on blood tests [19].

### Study population

**Fig. 1:**
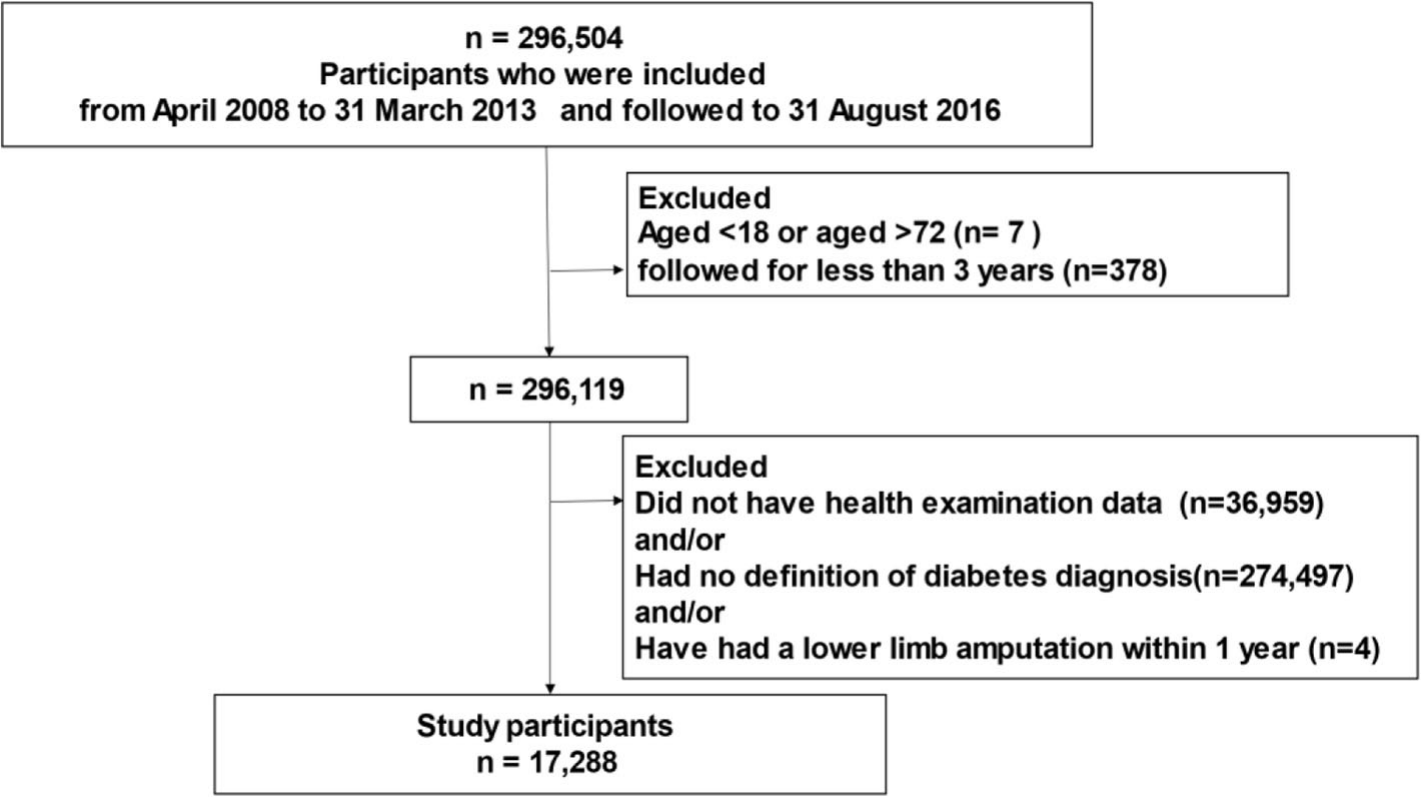
Flow chart for the extraction of study participants

People aged 18–72 years who had been followed for at least 3 years between 1 April 2008 and 31 March 2013 were included and followed up to 31 August 2016. For the present study, we examined data on 296,504 individuals. We then excluded 278,831 individuals who did not meet the definition of diabetes and/or required amputation within 1 month of enrollment and/or who had missing data. Finally, this study included 17,288 individuals with diabetes who had at least a 1-year amputation-free period before baseline (Fig. 1). The point of entry to the study was the earliest date for health examination data between 1 April 2008 and 31 March 2013. Mean age and HbA1c value were 50.2 years and 7.2%, respectively.

### Definitions

Diabetes was determined by fasting plasma glucose (FPG) ≥7.0 mmol/L or HbA1c ≥6.5% or both without prescription of an antidiabetic drug or with a prescription of an antidiabetic drug regardless of FPG or HbA1c. Blood pressure was measured at all participating facilities in accordance with the guidelines of the Japanese Society of Hypertension [20, 21]. For medical checkups, these guidelines recommended measuring blood pressure twice by the oscillometric method and averaging the results.

Occurrence of amputation was determined according to claims using Diagnosis Procedure Combination (Japanese original codes), the International Classification of Diseases 10th revision (ICD-10) codes and medical procedure data after 1 month of follow-up (Supplemental Table 1). Since we could not completely exclude patients with a past history of lower limb amputation, we used the term “rates of amputation”.

### Statistical analysis

Categorical variables were expressed as numerals and percentages. Continuous variables were expressed as the mean ± standard deviations (SD). For comparison between those with and without amputation, χ^2^ tests were used for the categorical variables. The unpaired Student’s *t* test or the Mann-Whitney U test based on distribution was used for continuous variables. Cox regression model identified variables related to amputation. First, we conducted a univariate analysis to determine the significant variable for amputation. Then, we performed a multivariate analysis using variables with *P* < 0.1 in the univariate analysis as covariates. We used categorical variables to clarify the impact of combinations of age and HbA1c on lower limb amputation.

Analyses were performed using SPSS (version 19.0, Chicago, IL, USA). Statistical significance was considered at *P* < 0.05. The Ethics Committee of the Niigata University approved this study.

## Results

Median and maximum follow-up periods were 5.3 and 8.4 years, respectively. During the study period, 16 study participants experienced a new amputation. The rate of amputation was 0.17 per 1000 person-years. Baseline characteristics of those who had or had not experienced amputation during the observational period are summarized in Table 1. Individuals with amputation were significantly older than those without amputation. The levels of HbA1c, systolic blood pressure (SBP), diastolic blood pressure (DBP) and the rate of current smoking were higher in patients with amputation compared to patients without amputation; however, no statistical differences were observed. The percentages of participants aged ≥60 years and with HbA1c ≥8.0% were significantly higher in patients with amputation compared to patients without amputation.

**Table 1:** Baseline characteristics of study participants according to the presence or absence of lower limb amputation

Characteristic cardiovascular manifestation in FH Kawaguchi et al. Am Heart J 1999	Lower limb amputation		*P*-value
	(−)	(+)	
	*n* = 17,272	*n* = 16	
Age (years)	50 ± 8	54 ± 10	0.025
≥60 (%)	2137(12)	6 (38)	0.002
Sex (men, %)	14,322 (83)	14 (88)	0.630
BMI (kg/m^2^)	26.1 ± 4.6	25.7 ± 5.4	0.768
SBP (mmHg)	130 ± 17	134 ± 25	0.556
DBP (mmHg)	80 ± 11	75 ± 14	0.050
HbA1c (%)	7.2 ± 1.4	8.3 ± 2.1	0.054
HbA1c IFCC (mmol/mol)	55 ± 16	67 ± 23	0.054
HbA1c ≥8.0 (%)	3542(21)	8 (50)	0.004
LDL-cholesterol (mmol/L)	3.3 ± 0.9	3.6 ± 1.5	0.449
HDL-cholesterol (mmol/L)	1.4 ± 0.4	1.4 ± 0.4	0.546
Current smoking (%)	6498 (38)	7 (44)	0.613

Data are presented as n (%), mean ± SD. *BMI* body mass index, *HbA1c IFCC* glycated hemoglobin, International Federation of Clinical Chemistry and Laboratory Medicine units, *SBP* systolic blood pressure, *DBP* diastolic blood pressure, *LDL-C* low-density lipoprotein-cholesterol, *HDL-C* high-density lipoprotein-cholesterol

**Fig. 2:**
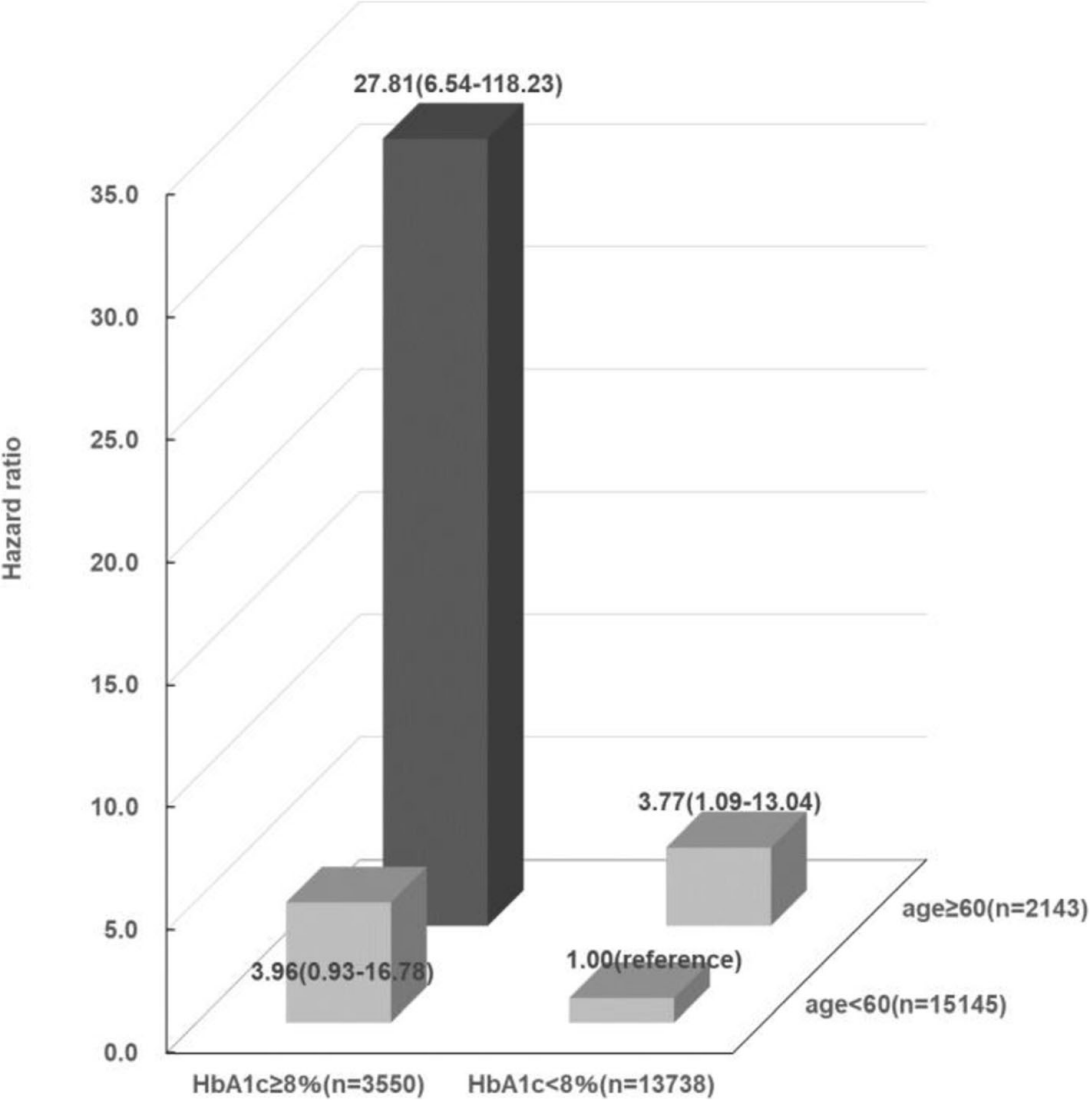
Hazard ratios for lower limb amputation according to combinations of HbA1c and age. The hazard ratio (HR) for those aged ≥60 years with HbA1c < 8.0% was 3.77 (95% confidence interval (CI) 1.09–13.04) and the HR for those aged < 60 years with HbA1c ≥8.0% was 3.96 (95% CI 0.93–16.78). The HR for those aged ≥60 years with HbA1c ≥8.0% was 27.81 (95% CI 6.54–118.23) compared with those aged < 60 years with HbA1c < 8.0% (reference group). Adjusted for diastolic blood pressure

Univariate Cox regression analysis showed that age (hazard ratio [HR] 1.08 [95% confidence intervals] 1.02–1.16, *p* = 0.01), DBP 5 mmHg increase (HR 0.79 (0.63–0.99), *p* = 0.04) and HbA1c (HR 1.38 [1.11–1.71], *p* < 0.01) were independently associated with amputation (Table 2). Multivariate Cox regression analysis showed that age (hazard ratio [HR] 1.09 [1.02–1.16], p < 0.01) and HbA1c (HR 1.46 [1.17–1.81], p < 0.01) were independently associated with amputation (Table 2). Figure 2 shows HRs according to age, HbA1c and their combinations for amputation analyzed by multivariate Cox models. Compared with those aged < 60 years with HbA1c < 8.0%, the HR was 3.77 (1.09–13.04) for those aged ≥60 years with HbA1c < 8.0%, 3.96 (0.93–16.78), for those aged < 60 years with HbA1c ≥8.0% and 27.81 (6.54–118.23) for those aged ≥60 years with HbA1c ≥8.0%.

**Table 2:** Cox regression analysis of variables associated with amputation

	Univariate analysis	*P*-value	Multivariate analysis	*P*-value
	HR (95% CI)		HR (95% CI)	
**Age (years)**	**1.08 (1.02–1.16)**	**0.01**	**1.09 (1.02–1.16)**	**0.01**
Sex	1.40 (0.32–6.17)	0.66	–	
BMI 1 kg/m^2^ increase	0.98 (0.88–1.10)	0.74	–	
SBP 10 mmHg increase	1.13 (0.86–1.47)	0.39	–	
**DBP 5 mmHg increase**	**0.79 (0.63–0.99)**	**0.04**	0.81 (0.64–1.01)	0.07
LDL-cholesterol 1 mmol/L increase	1.42 (0.85–2.37)	0.18	–	
HDL-cholesterol 1 mmol/L increase	0.67 (0.17–2.63)	0.56	–	
Current smoking	1.25 (0.46–3.35)	0.66	–	
**HbA1c (%)**	**1.38 (1.11–1.71)**	**< 0.01**	**1.46 (1.17–1.81)**	**< 0.01**

*BMI* body mass index, *DBP* diastolic blood pressure, *SBP* systolic blood pressure, *HR* hazard ratio, *CI* confidence interval, *LDL* low-density lipoprotein, *HDL* high-density lipoprotein, *HbA1c* glycated hemoglobin

## Discussion

In this study, we found that lower limb amputation frequency was low among young-adults to young-old Japanese people with diabetes. Age and HbA1c were associated with amputation. Compared with persons aged < 60 years and with HbA1c < 8.0%, the risk for amputation was significantly higher among those aged ≥60 years and with HbA1c ≥8.0%.

In USA, the most common causes of limb loss are diabetes with an age-adjusted incidence rate of 2.8 per 1000 person-years in people with diabetes [5]. In Taiwan, the trends for both major and minor lower limb amputation rates were decreased with incidence rates of 1.09 and 0.98 per 1000 person-years, respectively [22]. A recent study conducted in Japan showed that the incidence of diabetic foot ulcers and amputation were 2.9 and 0.47 per 1000 person-years, respectively [16]. In that study, the mean age was 65 years [16], which was much higher than in our study. In our study, the rate of amputation was 0.17 per 1000 person-years, which was extremely low compared to previous studies [16, 22]. These differences reflected differences in characteristics between study participants. For example, the mean age was younger in our study. Older patients may have a longer disease duration, higher number of comorbidities and poorer glycemic control, resulting in a higher incidence of amputation than in younger patients.

Adler et al. showed that there is a substantial increase in the risk of lower limb amputation associated with glycemia in individuals with diabetes [23]. In our multivariate analysis, HbA1c was independently associated with amputation. Interestingly, a recent meta-analysis showed that the HbA1c level does not affect the incidence of amputation in patients with diabetic foot ulcers [13]. Intensive control may decrease the risk of amputation in patients with diabetic foot syndrome [24]. Taken together, glycemic control may help reduce the frequency of lower limb amputations through reducing the development of diabetic foot ulcers, wound progression and progression of neuropathy.

Age plays an important role in atherosclerosis, resulting in an increased risk of cardiovascular disease. No clear association was observed between age and amputation in patients with diabetic foot ulcers [13]. We found that age was independently associated with lower limb amputation, which was consistent with a previous study [25]. Moreover, the risk for amputation was drastically higher among those aged ≥60 years and with HbA1c ≥8.0% compared with patients aged < 60 years and with HbA1c < 8.0% in our study of Japanese with diabetes. However, the sample size for stratified analysis of events was too small to conclude an association between HbA1c and lower limb amputation. Thus, future studies are needed to confirm our findings with an adequate sample size.

Hypertension was associated with both micro- and macro diabetic complications [26, 27]. Although hypertension is approximately twice as frequent in patients with than without diabetes [28], one third of persons with diabetes do not reach target blood pressure values [29]. Therefore, early and sustained blood pressure control is necessary to prevent not only cardiovascular disease but also lower limb amputation in clinical practice.

Our analysis did not show smoking as a significant risk factor for lower limb amputation. However, smoking remains a risk factor for cardiovascular events [30, 31]. Therefore, no conclusion can be drawn regarding the association between smoking and lower limb amputation from our study and future studies that stratify current, past and no smoking are needed.

BMI was not associated with lower limb amputation in this study. Chan et al. showed that the prevalence of obesity was higher in urban than in rural residents in Asia [32]. Generally, lower BMI was associated with increased mortality risk [33]. Unfortunately, we have no data as to whether a study participant resided in a rural or urban area. Another possibility is that our findings reflected an obesity paradox in patients with diabetes [34].

It was noted that men do not examine or look at their feet as often as women do, suggesting a lower level of foot care in men [35]. In our study, only two of the 16 amputations occurred in women. A meta-analysis showed that sex differences existed in the prevalence of glucose tolerance status [36]. However, in our univariate analysis, male sex was not associated with amputation. This may be due to the high proportion of males among both those with and without amputation in our cohort, which was different from both other cohorts and the national level in Japan [12] [37] [38]. Thus, our finding should be interpreted with caution and future studies of adequate populations are necessary to confirm the impact of sex differences on amputation among Japanese young adults to those classified as “young-old”.

This study’s strengths were the large sample size and accurate capture of diabetes diagnoses and lower limb amputation using data from health examinations, medical practice and a claims database. However, several limitations should be addressed. First, the sample size for amputation was too small to establish rates and predictors of amputation. Moreover, we do not have data on diabetic foot ulcers. Unfortunately, ICD-10 codes for diabetic foot ulcer are not commonly used in clinical settings in Japan. Therefore, we cannot accurately identify patients who had diabetic foot ulcers from claims data. Also, we could not distinguish between first, second and subsequent amputations from our database. However, we included patients who had least a 1-year amputation-free period before baseline using claims data. We attempted to distinguish between major and minor amputations. However, the number of amputations was too small to conduct a stratified analysis. Secondly, we have no data on details of smoking status and could not distinguish between never-smokers and former smokers. Thus, our findings should be interpreted with caution in regard to the impact of smoking on amputation. Thirdly, it was not possible to identify participants whose HbA1c, SBP or DBP had either improved or deteriorated during the follow-up period, as these values were measured at only one point in time. Fourth, we could not obtain information on the duration of diabetes, social factors and psychiatric factors that would affect the incidence of amputation. Fifth, the number of lower limb amputations may be underestimated due to coding errors.

## Conclusions

Age and HbA1c were associated with amputation. Compared with patients aged < 60 years and with HbA1c < 8.0%, the risk for amputation was much higher among those aged ≥60 years and HbA1c ≥8.0%, suggesting the need for ethnic group-specific strategies to prevent diabetic foot disease.

## Supplementary Information

**Additional file 1: Supplemental Table 1.** Definitions of amputation using claims.

## Data Availability

The data and study materials will not be made available to other researchers for purposes of reproducing the results or replicating the procedure.

## Abbreviations

DBP: Diastolic blood pressure
FPG: Fasting plasma glucose
HbA1c: Haemoglobin A1c
HRs: Hazard ratios
BMI: Body mass index
SBP: Systolic blood pressure
ICD-10: International Classification of Diseases 10th revision
