# Supplementary material for: Rates and risk factors for amputation in people with diabetes in Japan: a historical cohort study using a nationwide claims database
Source: J Foot Ankle Res. 2021 Apr 9;14:29. doi: 10.1186/s13047-021-00474-8 (PMC8034178; doi:10.1186/s13047-021-00474-8)
Supplement: Supplementary file 1 — Additional file 1: Supplemental Table 1. Definitions of amputation using claims. [file 13047_2021_474_MOESM1_ESM.docx]

**Supplemental Table 1. Definitions of amputation using claims**

| DPC code | ICD-10 code | procedure code |
| --- | --- | --- |
| 100100xx97x0xx, 100100xx97x1xx | E115, E 144,  E 145 | K0842, K0843, K0851, K0852, K0853 (excluding amputation of the upper limb) |

DPC: Diagnosis Procedure Combination; ICD-10; International Classification of Diseases: Tenth Revision
